# Comparison of the Effects of Desflurane, Sevoflurane, and Propofol on the Glottic Opening Area during Remifentanil-Based General Anesthesia Using a Supraglottic Airway Device

**DOI:** 10.1155/2020/1302898

**Published:** 2020-06-19

**Authors:** Takashi Kondo, Hiromichi Izumi, Makiko Kitagawa

**Affiliations:** ^1^Department of Anesthesiology and Critical Care, Hiroshima University Hospital, Hiroshima, Japan; ^2^Department of Anesthesia, Akane Foundation Tsuchiya General Hospital, Hiroshima, Japan

## Abstract

**Purpose:**

The aim of this study was to compare the effects of desflurane, sevoflurane, and propofol on the glottic opening area during general anesthesia using remifentanil.

**Methods:**

Ninety patients undergoing hand and upper limb surgery combined with brachial plexus block under general anesthesia were enrolled in the study. The patients were randomized into three groups to receive desflurane (group D), sevoflurane (group S), or propofol (group P) for maintenance of anesthesia. Following induction of general anesthesia with remifentanil, continuous fiberoptic video recording around the glottis via an i-gel™ supraglottic device was started after establishing mechanical ventilation. Desflurane, sevoflurane, or propofol was administrated after video recording was started. The changes in normalized glottic opening area (n-GOA) and peak inspiratory pressure (PIP) during surgery were compared between the three groups.

**Results:**

Intraoperative changes of n-GOA in group D showed significant differences compared with group S and group P (−0.0656 ± 0.0772 vs. −0.0076 ± 0.0499 and +0.0269 ± 0.0809, *P*=0.005 and *P* < 0.0001). The changes of PIP in group D showed significant differences compared with group S and group P (+3.7 ± 3.4 cmH_2_O vs. +1.0 ± 1.3 cmH_2_O and −0.3 ± 3.6 cmH_2_O, *P*=0.002 and *P* < 0.0001). Four cases of relapsed glottic stenosis in group D were improved by changing desflurane to propofol.

**Conclusions:**

Desflurane narrowed the n-GOA and increased the PIP compared to sevoflurane and propofol during general anesthesia with remifentanil. Clinicians should be aware of the possibility of glottic stenosis during desflurane-remifentanil anesthesia when the airway is secured by a supraglottic airway device without the use of neuromuscular blockade.

## 1. Introduction

Intraoperative vocal cord closure can lead to fatal complications, including hypoxia. Although vocal cord closure in children is commonly evoked by laryngospasm, in adults, both vocal cord closure and laryngospasm have been reported to be triggered by anesthetic agents such as opioids [1–4] and desflurane [5].

Propofol has been reported to decrease the occurrence of laryngospasm [6, 7], possibly by suppressing the laryngeal reflex [8]. However, the effects of anesthetic agents on the laryngeal muscles, including the vocal cords, during the maintenance of anesthesia under mechanical ventilation are still unclear. We have previously reported two cases of progressive intraoperative vocal cord closure during desflurane and remifentanil anesthesia at the appropriate depth of anesthesia, in which vocal cord closure was relieved after desflurane discontinuation and propofol administration [9]. The finding has shown that the glottic patency formed by vocal cords during mechanical ventilation could be different between desflurane and propofol.

In this study, we hypothesized that the glottic patency might be different between different anesthetics. Therefore, we compared the effects of desflurane, sevoflurane, and propofol on the glottic opening area during general anesthesia using remifentanil with mechanical ventilation.

## 2. Materials and Methods

After receiving approval for the study protocol from our institutional review board (Tsuchiya General Hospital) and obtaining written informed consent from potential participants, we enrolled 90 patients with an American Society of Anesthesiologists physical status of I or II who were to undergo elective hand and upper limb surgery combined with brachial plexus block under general anesthesia. Patients with the following problems were excluded from the study: preexisting oropharyngeal disease, severe obstructive sleep apnea, and morbid obesity (body mass index > 35 kg/m^2^). The patients were randomized into three groups to receive desflurane (group D), sevoflurane (group S), or propofol (group P) for maintenance of anesthesia by computer-generated randomization. The study was approved by the local ethics committee, and study protocols were registered in the University Hospital Medical Information Network (UMIN, ID: UMIN000009164).

### 2.1. Video Recording of the Glottis during General Anesthesia

Following the induction of general anesthesia with 0.5 *μ*g/kg/min of remifentanil, 5 mg/kg of thiopental, or 0.05 mg/kg of midazolam (in patients with a history of asthma), as well as 0.06 mg/kg of rocuronium for prevention of muscle rigidity caused by remifentanil [10], an i-gel™ supraglottic device was inserted after the bispectral index (BIS) value fell below 60. After establishing volume-controlled mechanical ventilation with a tidal volume of 8 ml/kg and a fresh gas flow of 6 L/min, a 5 mm fiberscope was placed into the i-gel™ via a swivel port connector, and then continuous video recording around the glottis was begun. After that, desflurane, sevoflurane, or propofol was started according to the predetermined patient group and titrated to achieve a BIS value of 40–60. Patients were excluded if the BIS value increased over 60. A continuous infusion of remifentanil was titrated to around 0.2 *μ*g/kg/min. Rocuronium was not added after induction. An ultrasound-guided axillary brachial plexus block was performed after addition of the three anesthetics. If mechanical ventilation became difficult during surgery, an attending anesthesiologist was allowed to increase the anesthetic doses if insufficient depth of anesthesia was suspected. When the depth of anesthesia was appropriate, an attending anesthesiologist was allowed to add rocuronium or change the anesthetic type. Video recording was continued until the surgery was finished.

### 2.2. Analysis of the Glottal Area

The primary outcomes of this study were to compare the intraoperative changes of the glottic opening area (GOA) during mechanical ventilation between the three anesthetic agents. The changes of the peak inspiratory pressure (PIP) after starting the three anesthetics were also compared.

After the surgery, an investigator (the corresponding author) analyzed the recorded images. The GOA at the opening phase was calculated at two points, as follows: GOA1, defined as the baseline value obtained before starting the three anesthetic agents, and GOA2, defined as the intraoperative value measured at the moment of maximum change from baseline. The GOA was calculated using the area-tracing software AREA-Q (S-tech, Chiba, Japan).

**Figure 1:**
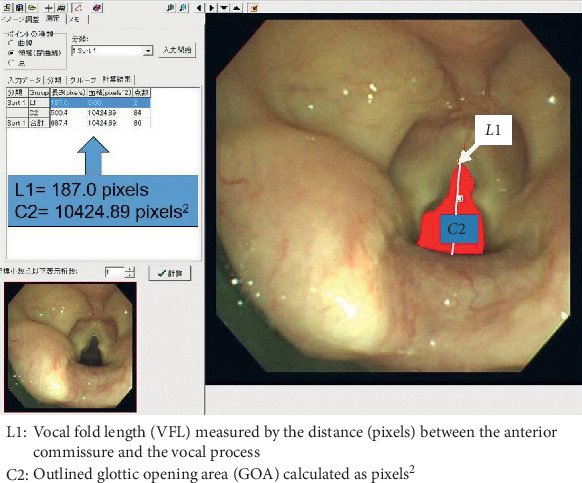
Imaging of the glottis and calculation of the glottic opening area (GOA).

An example of the calculated GOA is shown in Figure 1. First, the vocal fold length (VFL) was measured by the distance (pixels) between the anterior commissure and the vocal process, and the outlined GOA was calculated as pixels^2^. Second, the GOA was normalized (n-GOA) by calculating GOA/VFL^2^ with reference to a previous report [11]. The changes in n-GOA from baseline expressed as (n-GOA2-n-GOA1) were compared between groups D, S, and P. The peak inspiratory pressure (PIP) was also recorded at the same time points defined by GOA1 and GOA2 and designated as PIP1 and PIP2, respectively. The changes in PIP from baseline expressed as (PIP2-PIP1) were compared between the three groups.

### 2.3. Patient Background Factors

The following patient demographic and clinical characteristics were investigated in this study: age, gender, height, body weight, vital capacity as percent of predicted, forced expiratory volume percent in 1 second, history of asthma, smoking, mean dose of remifentanil, mean doses of anesthetics for maintenance, train-of-four ratio of the adductor pollicis muscle before inserting i-gel™, surgical time, and anesthesia time.

### 2.4. Statistical Analyses

All data were analyzed using PRISM 8.0 software (GraphPad Software, San Diego, CA, USA). All background factors and related factors were compared using one-way ANOVA and Pearson's chi-squared test. Values and changes of n-GOA and PIP were compared using one-way ANOVA and repeated-measures ANOVA. The Tukey–Kramer test was used for post hoc analysis to compare differences among groups. *P* values <0.05 were considered to be statistically significant. Although we could not calculate the optimal sample size before the study, we confirmed that it was appropriate by calculating effect sizes for comparisons of changes in n-GOA and PIP. The effect size was calculated by dividing the *Z*-score of a standard normal distribution by the square root of the sample size. *G*^*∗*^power 3.1.0 program (http://www.gpower.hhu.de/) was used to calculate the required effect size. To obtain a 0.80 power between the groups, the required effect size was calculated as 0.40.

## 3. Results

**Figure 2:**
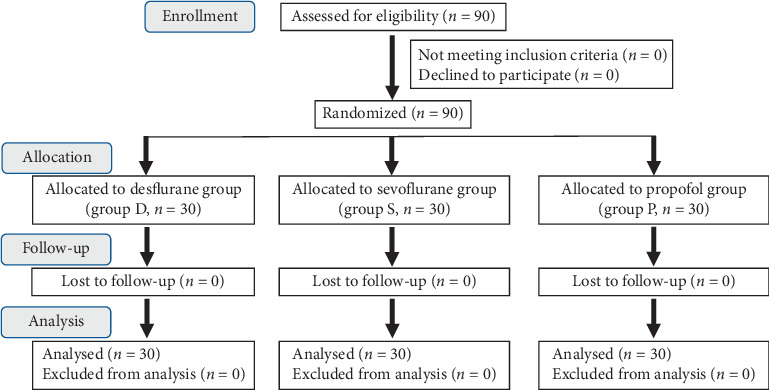
The CONSORT diagram.

The CONSORT diagram is shown in Figure 2. Patient demographic and clinical characteristics were similar between the three groups (Table 1). There were no cases of failed i-gel™ insertion, no patients with BIS values > 60, and no body movements or indications of spontaneous breathing during the surgery.

**Figure 3:**
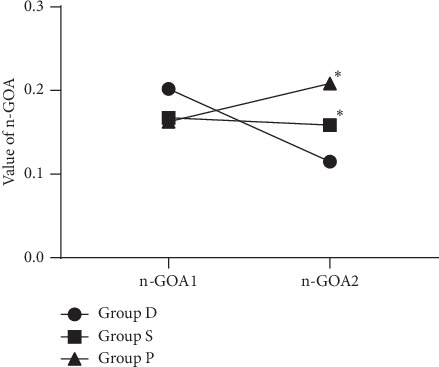
The changes of normalized glottic opening area (n-GOA) from baseline after administration of desflurane (group D), sevoflurane (group S), and propofol (group P). Data are shown as mean value. ^*∗*^*P* < 0.05 vs. group D in the change of n-GOA.

Intraoperative changes in n-GOA are shown in Table 2. There were no significant differences in n-GOA baseline values between the three groups. The differences between the three groups were statistically significant (Figure 3, *P* < 0.0001). In a post hoc analysis, the changes of n-GOA (n-GOA2-n-GOA1) in group D were significantly larger than that in group S (*P*=0.005, an effect size of 0.44) and that in group P (*P* < 0.0001, an effect size of 0.61).

Intraoperative changes in PIP are shown in Table 3. There were no significant differences in PIP baseline values between the three groups.

**Figure 4:**
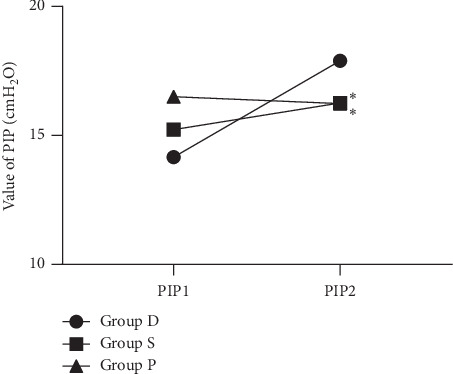
The changes of peak inspiratory pressure (PIP) from baseline after administration of desflurane (group D), sevoflurane (group S), and propofol (group P). Data are shown as mean value. ^*∗*^*P* < 0.05 vs. group D in the change of PIP.

**Figure 5:**
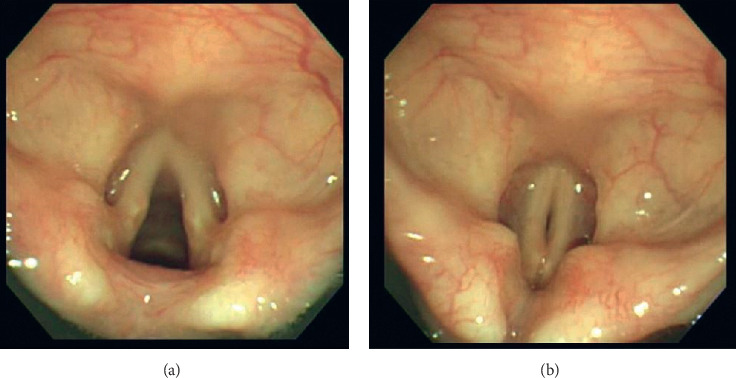
Typical example of the narrowed glottal area during desflurane-remifentanil anesthesia: (a) before desflurane administration and (b) after desflurane administration.

The differences between the three groups were statistically significant (Figure 4, *P* < 0.0001). In a post hoc analysis, the changes of PIP (PIP2-PIP1) in group D were significantly larger than that in group S (*P*=0.002, an effect size of 0.47) and in group P (*P* < 0.0001, an effect size of 0.62). Six patients in group D required treatment for glottic stenosis with increased PIP. The n-GOA2 of the six patients was 0.0555 ± 0.0395. All six patients were first treated by the addition of rocuronium; however, glottic stenosis recurred after the effect of rocuronium disappeared. In four of the six patients, the recurrence improved after desflurane was changed to propofol.  Figure 5 shows a typical example of the narrowed glottal area during desflurane-remifentanil anesthesia.

## 4. Discussion

In this study, endoscopic video recording during remifentanil-based general anesthesia indicated that the glottal area varied according to the type of anesthetic coadministered with remifentanil. Desflurane narrowed the n-GOA more than sevoflurane and propofol. The change in PIP was significantly larger during desflurane-remifentanil anesthesia than during sevoflurane-remifentanil anesthesia and propofol-remifentanil anesthesia. Additionally, propofol was useful to improve intraoperative glottic stenosis during desflurane-remifentanil anesthesia.

Intraoperative glottic stenosis results from increased laryngeal muscle tone, such as that occurring during laryngospasm. Laryngospasm is often the result of the laryngeal reflex elicited by stimulation of the superior laryngeal nerve, which is the afferent pathway of the laryngeal adductor muscles [12]. Laryngospasm can be partial [13], in which case the bilateral vocal cords do not close completely. Insufficient depth of anesthesia is a common cause of laryngospasm during the perioperative period. In this study, there were no findings of insufficient depth of anesthesia, such as BIS values > 60 or body movement or appearance of spontaneous breathing during surgery. Additionally, ultrasound-guided brachial plexus block was successfully performed in all cases. Therefore, the depth of anesthesia was considered to be appropriate in all cases. Although the exact pathophysiological mechanism of laryngospasm during general anesthesia remains unclear, several anesthesia-related factors have been identified.

Administration of opioids can lead to laryngospasm, since they increase muscle tone and thereby cause muscle rigidity, including in the laryngeal muscles [1]. Instances of opioid-induced vocal cord closure have been reported in surgical cases. Fodale et al. [3] successfully treated tramadol-induced vocal cord closure with naloxone. Furthermore, animal studies have shown that activation of the central *μ*-opioid receptor plays an important role in opioid-induced muscle rigidity [14], which commonly occurs during anesthesia induction due to a rapid increase in the concentration of the administered opioid. By contrast, we observed vocal cord closure during the maintenance phase of anesthesia, in which remifentanil was administered at a dosage of 0.15–0.2 *μ*g/kg/min. The vocal cords did not close before starting desflurane, sevoflurane, or propofol in this study, which may indicate that closure in our cases was not caused by remifentanil.

The use of volatile anesthetics, especially desflurane, increases the incidence of laryngospasm, partially by stimulating airway receptors during anesthesia induction. For instance, transient receptor potential A1 (TRPA1) in the upper airway responds in a dose-dependent manner to the sympathetic activation that accompanies rapid increases in the concentration of desflurane [15–17]. On the other hand, the incidence of laryngospasm during maintenance of anesthesia under mechanical ventilation has not been well documented. A study using a laryngeal mask airway in spontaneously breathing patients showed that a concentration of desflurane under 6% (1.0 minimum alveolar concentration [18]) enhanced airway reactivity and increased respiratory complications in both smokers and nonsmokers in comparison with sevoflurane [19, 20]. Therefore, even during the maintenance phase of anesthesia with a low concentration of desflurane, airway reflex activation can occur, causing laryngospasm.

By contrast, propofol has been shown to be useful for the treatment of laryngospasm both during the postextubation period [6] and after removal of a laryngeal mask [7]. The effect of propofol on intraoperative laryngospasm is not well known, although it has been reported to decrease the occurrence of laryngospasm elicited by spraying distilled water via a laryngeal mask airway [8]. Although the mechanism is unclear, propofol may suppress glottal stenosis due to the laryngeal reflex.

In this study, an i-gel™ supraglottic airway device was used to secure the airway. Such devices do not prevent glottic stenosis; therefore, vocal cords may close during surgery. Under mechanical ventilation, desflurane-remifentanil anesthesia narrowed the GOA and increased airway pressure more than sevoflurane-remifentanil anesthesia and propofol-remifentanil anesthesia. Our findings indicate that attention should be paid to the possibility of glottic stenosis during desflurane-remifentanil anesthesia when the airway is secured by a supraglottic airway device without the use of neuromuscular blockade.

## Figures and Tables

**Table 1:** Patient demographic and clinical characteristics.

	Group D	Group S	Group P	*P* value
*n*	30	30	30	
Age (y)	50 ± 18	50 ± 17	49 ± 17	0.9556
Male : female	19 : 11	17 : 13	18 : 12	0.8703
Height (cm)	165 ± 9	163 ± 9	165 ± 10	0.6887
Body weight (kg)	65 ± 12	61 ± 12	66 ± 14	0.3258
% VC (%)	107 ± 20	111 ± 17	114 ± 19	0.4069
FEV1% (%)	86 ± 8	85 ± 6	84 ± 6	0.4055
History of asthma	1	1	1	>0.9999
Current smoker	5	3	6	0.5532
Mean remifentanil dose (*μ*g/kg/min)	0.19 ± 0.03	0.21 ± 0.04	0.20 ± 0.04	0.2826
Mean dose of maintenance anesthetics	4.8 ± 1.3 (%)	1.6 ± 0.3 (%)	2.2 ± 0.3 (*μ*g/ml)	—
Train-of-four ratio before inserting i-gel™ (%)	94 ± 23	90 ± 11	96 ± 13	0.3904
Surgical time (min)	164 ± 78	158 ± 60	160 ± 81	0.9487
Anesthesia time (min)	225 ± 82	223 ± 65	229 ± 89	0.9798

VC: vital capacity; FEV: forced expiratory volume. Usage of propofol is expressed as target-controlled infusion. Data are shown as mean ± SD.

**Table 2:** Values of normalized glottic opening area (n-GOA) in the three anesthesia groups.

	Group D	Group S	Group P	*P* value
n-GOA1	0.2172 ± 0.0724	0.1971 ± 0.0882	0.1921 ± 0.093	0.482
n-GOA2	0.1517 ± 0.0906	0.1895 ± 0.0929	0.219 ± 0.0623	0.009

GOA1: baseline value before starting each of the three anesthetic agents (desflurane: group D; sevoflurane: group S; propofol: group P); GOA2: the intraoperative value measured at the moment of maximum change from baseline. Data are shown as mean ± SD.

**Table 3:** Value of peak inspiratory pressure (PIP) in the three anesthesia groups.

	Group D	Group S	Group P	*P* value
PIP1	14.2 ± 2.9	15.2 ± 3.7	16.5 ± 4.7	0.0673
PIP2	17.9 ± 3.3	16.3 ± 3.8	16.2 ± 3.2	0.109

PIP1: baseline value before starting each of the three anesthetic agents (desflurane: group D; sevoflurane: group S; propofol: group P); PIP2: the intraoperative value measured at the moment of maximum change from baseline. Data are shown as mean ± SD.

## Data Availability

The data used to support the findings of this study are available from the corresponding author upon request.
